# Regulation of chaperone function by coupled folding and oligomerization

**DOI:** 10.1126/sciadv.abc5822

**Published:** 2020-10-21

**Authors:** Guillaume Mas, Björn M. Burmann, Timothy Sharpe, Beatrice Claudi, Dirk Bumann, Sebastian Hiller

**Affiliations:** Biozentrum, University of Basel, Klingelbergstrasse 70, 4056 Basel, Switzerland.

## Abstract

The homotrimeric molecular chaperone Skp of Gram-negative bacteria facilitates the transport of outer membrane proteins across the periplasm. It has been unclear how its activity is modulated during its functional cycle. Here, we report an atomic-resolution characterization of the *Escherichia coli* Skp monomer-trimer transition. We find that the monomeric state of Skp is intrinsically disordered and that formation of the oligomerization interface initiates folding of the α-helical coiled-coil arms via a unique “stapling” mechanism, resulting in the formation of active trimeric Skp. Native client proteins contact all three Skp subunits simultaneously, and accordingly, their binding shifts the Skp population toward the active trimer. This activation mechanism is shown to be essential for *Salmonella* fitness in a mouse infection model. The coupled mechanism is a unique example of how an ATP-independent chaperone can modulate its activity as a function of the presence of client proteins.

## Teaser

Trimeric chaperone Skp activates by an unprecedented mechanism, with folding from a disordered state coupled to trimerization.

## INTRODUCTION

Molecular chaperones are central for the survival of the cell in all kingdoms of life (*1*–*2*). They are involved in many cellular processes such as helping proteins to fold, preventing protein aggregation, and reducing cellular stress (*3*). Some chaperones can use adenosine triphosphate (ATP) binding and hydrolysis to trigger conformational changes that, in turn, regulate their functional cycle, including their interaction with client proteins (*4*). ATP-independent chaperones, in turn, lack this possibility. Nonetheless, some ATP-independent chaperones were found to be regulated by major conformational changes, and the transition mechanisms for the activation of ATP-independent chaperones have been classified into three categories (*5*): oligomer disassembly [small heat shock protein (sHSP) (*6*) and trigger factor (TF) (*7*–*9*)], order-to-disorder transition {Hsp33 (*10*), HdeA [HNS (histone-like nucleoid structuring)–dependent expression A] (*11*), and HdeB (*12*)}, and lack of major conformational change [spheroplast protein Y (Spy) (*13*, *14*), seventeen kilodalton protein (Skp) (*15*), HSP40 (*16*), SecB (*17*), and survival factor A (SurA) (*18*)]. These mechanisms of activation are of major biological importance, because constitutively active chaperones can interfere with protein folding processes and proteostasis due to their high affinity and low specificity for client proteins, thus representing a potential hazard to cells (*19*–*22*). An example of these detrimental effects has been reported for a constitutively active variant of the chaperone Hsp33, which lead to accumulation of large amounts of insoluble aggregates and severe growth disadvantages (*20*).

Representative of the first category, binding of chaperone sHSP to its client proteins is regulated via a shift from an inactive oligomeric ensemble toward an ensemble of smaller multimers, representing the active species (*6*). The monomeric species exposes a binding motif that is shielded within the oligomeric structure, making the large oligomeric state an inactive storage form that can be activated upon dissociation (*23*, *24*). Similarly, it has been shown that binding of TF to client proteins is accompanied by a shift from the inactive dimeric state toward the active monomeric state (*7*–*9*). By contrast, the order-to-disorder activation is found for chaperones where the active form is intrinsically disordered. Thereby, to shift from the folded inactive chaperone to the unfolded active chaperone, not only the oligomeric state but also the secondary structure of the chaperone is undergoing change, triggered either by a pH drop to acidic conditions (HdeA and HdeB) or by a redox transition (Hsp33). Once stress factors are reduced, these chaperones can return to their folded/oligomeric inactive state with a release of the client (*25*). The third category contains chaperones for which only one conformational state is known, and therefore, these are assumed to require no major conformational changes for their activation, as well as chaperones for which activation requires only minor conformational changes. One such example is provided by the chaperone Hsp40, which has minimal structural differences between its client-bound and apo state (*16*). Another example is given by the chaperone SecB, for which high-resolution structures of client-bound states showed only a minor conformational change to the inactive client-free state (*17*). In the client-free form, helix α2 acts as a lid of the client protein binding site. Upon client binding, this helix swings outward, thereby allowing access to the client binding groove. Similarly, the chaperone SurA has been shown to have a dynamic mechanism of activation where a domain connected to the chaperone core by linkers assists client protein recognition, binding, and release (*18*).

The periplasmic chaperone Skp is an integral part of outer membrane protein (Omp) biogenesis, on a parallel pathway with the chaperone SurA. Skp transports Omps in their unfolded state across the periplasm toward their insertion point into the outer membrane (*26*–*28*). *Yersinia skp* and *Salmonella skp* mutants show compromised virulence in rodent infection models, indicating a crucial role of Skp in vivo (*29*, *30*). Skp is structurally characterized by a trimeric oligomeric state with a “jellyfish”-like architecture (*31*, *32*). Each protomer contributes three β-strands toward a nine-stranded β-barrel in the trimerization interface and a long, α-helical “arm,” made of two α-helices in coiled-coil arrangement (*31*, *32*). The combination of three arms from the individual subunits leads to formation of a cavity that can accommodate and bind unfolded Omps (*15*, *33*).

The elongated arms of Skp are highly flexible in the apo state, and a recent molecular dynamics study has identified a pivot element to act as a hinge, allowing Skp to adapt to clients of different sizes (*15*, *34*). Upon binding, the Skp arms undergo a rigidification and keep the bound Omps inside the cavity in the “fluid globule” state (*15*, *35*). While Skp can accommodate differently sized protein clients, all functional complexes observed so far feature an Omp:Skp stoichiometry of 1:3 or 1:6, depending on the size of the client, suggesting that Skp binds clients always as a trimer (*36*). A recent study has emphasized that at physiological concentrations, Skp exists as an equilibrium between a trimeric and a monomeric form (*37*). The equilibrium was quantified by analytical ultracentrifugation (AUC), showing that the monomeric form is strongly dominant at 2 μM Skp, the concentration found in *Escherichia coli* stationary phase (*38*, *39*). The monomeric form of Skp has been proposed to be well folded based on indirect evidence (*37*); however, it has so far not been possible to directly analyze its structure, because at the high concentration required for most biophysical methods, the protein is mostly trimeric. Consequently, the structural features of the Skp monomeric state and the Skp activation mechanism remain poorly understood.

Here, we bypass this analytical challenge by introducing several weakly and non-oligomerizing mutants of Skp. We characterize their monomeric states by solution nuclear magnetic resonance (NMR) spectroscopy at the atomic level. The emerging reference data can then be used to fruitfully understand monomeric Skp(WT). The data show that monomeric Skp is intrinsically disordered and inactive and that binding of a client protein triggers Skp trimerization and activation. Last, we demonstrate that this mechanism is essential for bacterial virulence under in vivo conditions in a mouse infection model. The data thus reveal an essential mechanism regulating Skp chaperone activity by a combined disorder-to-order and oligomerization transition.

## RESULTS

### Design of monomeric Skp mutants

**Fig. 1:**
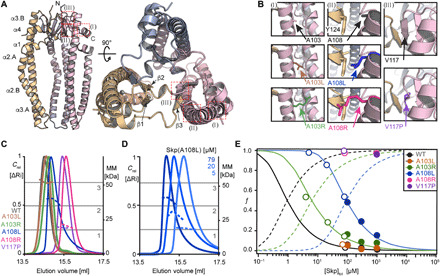
Identification of Skp mutations that perturb the monomer-trimer equilibrium. (**A**) Location of the mutation sites [red boxes (I), (II), and (III)], displayed on the Skp crystal structure (Protein Data Bank: 1SG2). Secondary structure elements and termini are indicated. (**B**) Close-up of the interface between Skp subunits, highlighting the position of the five mutations. See text for details. (**C**) SEC elution profiles (solid lines, left axis) and MALS apparent molecular mass (MM) (dotted lines, right axis) at elution concentrations of ≃80 μM and a temperature of 25°C. Dark gray, Skp(WT); brown, Skp(A103L); green, Skp(A103R); blue, Skp(A108L); magenta, Skp(A108R); purple, Skp(V117P). Gray horizontal lines indicate the molecular masses of monomers, dimers, and trimers of Skp. (**D**) Experiment as in (C) for Skp(A108L) as a function of the elution concentrations: 5, 20, and 79 μM. (**E**) Fractional populations *f* of monomers in the monomer-trimer equilibrium as a function of total Skp concentration. Experimental data points from NMR and SEC-MALS are indicated by filled and open circles, respectively. These have been fitted by Eq. 4 for mutants A103R and A108L (solid lines). The corresponding fractional populations of the trimeric state, 1 − *f*, are shown by dashed lines. Note that the concentration of Skp trimers equals one-third of the concentration of Skp molecules in the trimeric state, i.e., [Skp]_trimer_ = 1/3 · (1 − *f*) · [Skp]_tot_. For Skp(WT) and Skp(A103L), the data follow the WT association constant published by Sandlin *et al.* (*37*).

To prepare samples of monomeric Skp at concentrations sufficient for structural characterization, we set out to design mutants that would destabilize the oligomerization interface to shift the oligomerization equilibrium toward the monomeric form. The structure of trimeric Skp is stabilized by a network of three β-sheets per subunit that together form the trimerization interface in the “head” of the molecule (Fig. 1A). We identified the conserved alanine-103 and alanine-108 as promising candidates, because they are located at the oligomerization interface with limited space for their side chains. Their replacement by a bulkier side chain such as leucine or arginine should introduce steric clashes, leading to destabilization of the trimer (Fig. 1, A and B). In addition, we designed the mutant V117P to insert a proline residue, which is a known secondary structure breaker, into the trimerization β-sheet (β2). The oligomerization state of each of the Skp mutants Skp(A103L), Skp(A103R), Skp(A108L), Skp(A108R), and Skp(V117P) was determined by SEC-MALS (size-exclusion chromatography coupled to multi-angle light scattering) experiments at an elution concentration of ≃80 μM. At this concentration, the wild-type (WT) protein is mostly trimeric with a monomeric fraction lower than 4%. The mutant A103L was hardly distinguishable from WT, but the other Skp variants featured a gradually increased monomeric fraction, as evidenced by a smaller apparent mass, in the order A103R, A108L, V117P, and A108R (Fig. 1C). Thereby, mutants A108R and V117P were fully monomeric, and the others had effective molecular weights in between monomer and trimer, suggesting the presence of dynamic equilibria. We quantified the concentration dependence of these equilibria for Skp(A103L), Skp(A103R), and Skp(A108L) by solution NMR spectroscopy and SEC-MALS experiments (Fig. 1, C to E; Table 1; and fig. S1). Skp(WT) followed an equilibrium with *C*_0.5_ = 1.5 μM, the protein concentration at which half of the molecules are in the monomeric form, in agreement with published data (*37*). Skp(A103L) showed a trimer-monomer equilibrium that was essentially identical to WT (Fig. 1E and fig. S1), whereas Skp(A103R) had *C*_0.5_ = 7 ± 2 μM and Skp(A108L) had *C*_0.5_ = 80 ± 20 μM, indicating that these mutations shifted the equilibrium by about one to two orders of magnitude toward the monomer (Fig. 1, D and E, and fig. S1). The two mutants Skp(A108R) and Skp(V117P) were found to be monomeric at concentrations of even up to 1 mM (Fig. 1E and fig. S1).

**Table 1:** Biophysical and in vivo properties of Skp variants. Error estimates have been omitted for clarity. n.d., not determined.

	***C*_0.5_ (μM)**	**Skp activity (% of** **WT)**	**Trimer (%) at** ***c* ≃ 80 μM**	**Monomer (%) at** ***c* ≃ 80 μM**	***Salmonella* fitness** **in rich LB (% of** **WT)**	***Salmonella* fitness** **in mouse infection** **model (% of WT)**
Skp(WT)	1.5	100	97	3	100	100
Skp(A103L)	1.5	100	94	6	106	92
Skp(A103R)	7	87	86	14	102	58
Skp(A108L)	80	55	47	53	n.d.	n.d.
Skp(A108R)	>1000	1	0	100	n.d.	n.d.
Skp(V117P)	>1000	1	0	100	102	54
ΔSkp	–	–	–	–	104	54

We then characterized the structural integrity of Skp(A103L), Skp(A103R), and Skp(A108L) in their trimeric forms by NMR spectroscopy. For each of these proteins, two-dimensional (2D) [^15^N,^1^H]-TROSY (transverse relaxation-optimized spectroscopy) fingerprint spectra show the presence of two species in slow exchange on the NMR time scale, i.e., with kinetic exchange rate constants *k*_ex_ ≤ 10 s^−1^ (fig. S1). For each of the mutants, the overlay of the NMR spectra at 25°C (fig. S2) shows a high degree of similarity with the WT protein for most resonances, with considerable chemical shift perturbations only for some residues. Those residues are all located in spatial vicinity of the mutation site, in full agreement with the expected local distortion effects of single point mutations (fig. S2). The signals of residues located in the arms are not affected by the mutations, suggesting that symmetry and structural integrity of the trimeric form of the protein are maintained in the mutant. Oligomeric states other than the monomer and the trimer were not detected. The mutations thus shift the oligomerization equilibrium while leaving the trimeric form largely intact.

### Monomeric Skp is intrinsically disordered

**Fig. 2:**
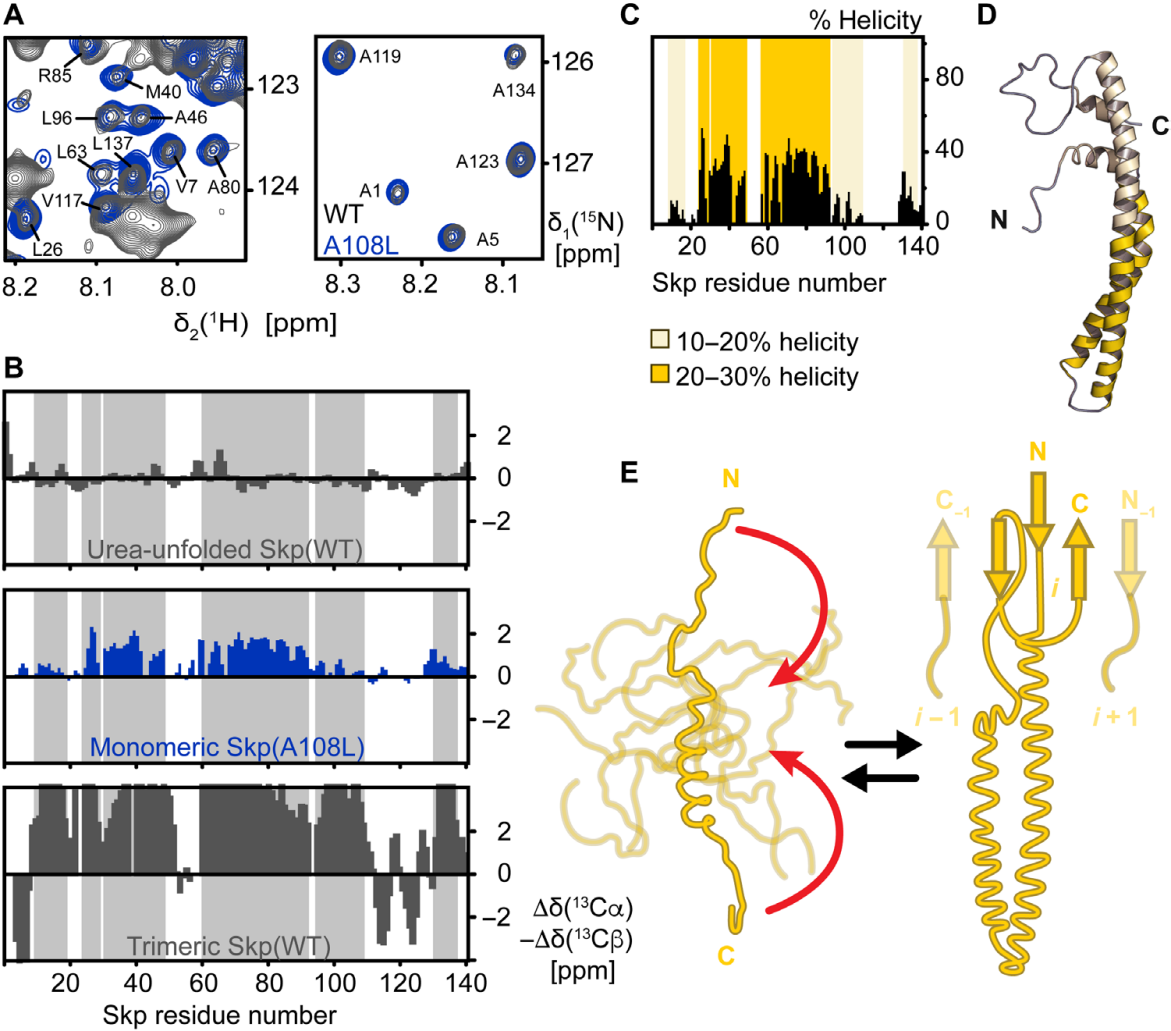
Monomeric Skp is intrinsically disordered. (**A**) Sections of 2D [^15^N,^1^H]-TROSY spectra of [*U*-^2^H,^15^N] Skp(WT) (dark gray) and Skp(A108L) (blue) at a concentration of 1 mM and 37°C in NMR buffer (20 mM MES, pH 6.5, and 150 mM NaCl). NMR signals of the monomeric state of Skp(WT) are overlaying with the one from Skp(A108L). The assignments of the overlapping NMR signals of the monomeric state are indicated in the panel. (**B**) Residue-specific secondary backbone chemical shifts of Skp(WT) in 8 M urea solution, Skp(A108L) in its monomeric form, and Skp(WT) in its trimeric form. Positive and negative values indicate α-helical and β-sheet secondary structure elements, respectively. The gray-shaded area indicates the positions of helices in the Skp trimer. (**C**) Percentage of helical population in the conformational ensemble of the Skp monomer. Helical regions with 10 to 20% helicity or 20 to 30% helicity are highlighted with light or dark yellow, respectively. (**D**) Structural model of the Skp monomer. On a configuration of Skp with α-helices formed, the degree of residual helical population present in the conformational ensemble is indicated. The large majority of monomeric Skp is disordered. (**E**) Schematic model of coupled oligomerization and folding mechanism of Skp. Monomeric Skp explores an ensemble of conformations with a low propensity for the formation of the arm α-helices. The formation of the oligomerization interface brings the N and C termini together (red arrows), thus stabilizing the coiled-coil structure of the α-helical arms.

The mutant Skp(A108L) with a *C*_0.5_ of 80 ± 20 μM at 25°C allowed us to prepare the monomeric state at concentrations of 100 μM and above, which is required for solution NMR spectroscopy. The NMR spectra of monomeric Skp(A108L) are completely overlapping with the monomeric, but low-abundant conformation of Skp(WT) (Fig. 2A), indicating that the conformations are essentially identical and thus validating the further analysis. Increasing the temperature from 25° to 37°C shifted the equilibrium of Skp(A108L) further toward the monomer, resulting in around 95% monomer at a concentration of 1 mM and thus further increasing the NMR signal intensity (fig. S2). A primary classification of the type of conformational state of monomeric Skp was obtained from the observation of a narrow chemical shift dispersion of backbone amide NMR signals, which is characteristic for proteins with low structural propensity (fig. S3). To quantify the secondary structure elements, we established complete sequence-specific resonance assignments of the monomeric state (fig. S3) and determined backbone ^13^Cα and ^13^Cβ secondary chemical shifts (Fig. 2B). These show that the three β-sheets that constitute the oligomerization interface in the trimeric form are in random-coil conformation in the monomeric state. Furthermore, the four α-helices forming the arms of Skp are in a fast conformational exchange between folded and unfolded conformations, as evidenced by the observation of a single set of resonances in fast exchange. Taking the fully denatured form of Skp in 8 M urea solution and the folded trimer as reference points, the residual helicity can be quantified for each residue (Fig. 2, B and C). The analysis shows that the helices α1, α3.B, and α4, which are closest to the trimerization interface, feature a residual helicity of <20%, while the helices α2.A, α2.B, and α3.A located at the tip of the arms display a helical population of 20 to 30% (Fig. 2, C and D). Overall, these data show that a small amount of residual α-helical structure is present in the disordered Skp monomers, but that the complete formation of the helices requires the trimerization interface. Overall, these data demonstrate that the monomeric state of Skp is intrinsically disordered with some residual helical propensity located at the tip of the arms. In the trimeric structure, the circular-barrel interface, connecting the N- and C-terminal part of the protein, brings helices α2 and α3 close together in space and thus stabilizes their secondary structure (Fig. 2E). This unique mechanism resembles a “stapling” of the coiled-coil helices to the barrel in the head domain.

It has been previously proposed that the monomeric state of Skp would be well folded rather than disordered (*37*). That conclusion was obtained from indirect measurements of the molar heat capacity change Δ*C*_p_ of trimer formation by a van’t Hoff analysis of temperature-dependent AUC data, which indicated a value of Δ*C*_p_ = −0.62 ± 0.11 kcal mol^−1^ K^−1^ for the Skp monomer-trimer transition. Because the authors expected a value for a coupled folding and oligomerization of Δ*C*_p_ = −8.01 ± 3.3 kcal mol^−1^ K^−1^, they concluded that only trimerization, but not folding, would take place during oligomerization. To resolve these different views, we determined Δ*C*_p_ of Skp(WT) directly by differential scanning calorimetry (DSC) to Δ*C*_p_ = −2.9 ± 0.4 kcal mol^−1^ K^−1^ at 37°C (fig. S3). Considering the average residual helicity of 21% in the monomer, this corresponds to a value of −1.1 kcal mol^−1^ K^−1^ for folding of one monomer subunit, which is a similar value to proteins of the same size (*40*, *41*). We note that Δ*C*_p_ is strongly temperature dependent (fig. S3), which may have perturbed the precision of the van’t Hoff analysis by Sandlin *et al.* (*37*).

### Omp binding stabilizes the functional Skp trimer

**Fig. 3:**
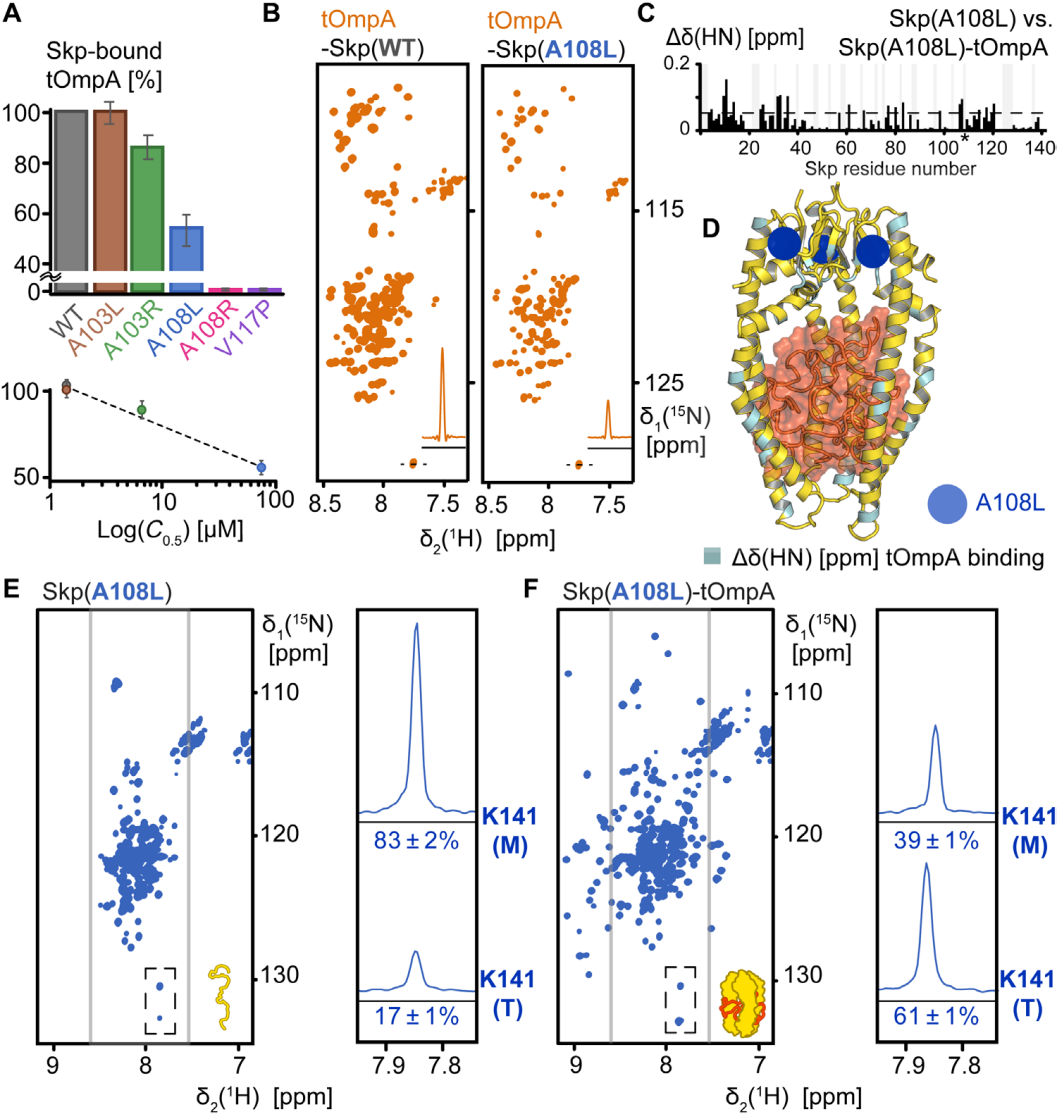
Client binding promotes Skp oligomer assembly. (**A**) Holdase activity of Skp variants as determined by the amount of aggregation-prone tOmpA solubilized in equilibrium. Values are normalized to the activity of Skp(WT). Error bars represent the SD of 15 individual signals of tOmpA. (**B**) 2D [^15^N,^1^H]-TROSY fingerprint spectra of [*U*-^2^H,^15^N]-tOmpA bound to unlabeled Skp(WT) or Skp(A108L). Spectra were recorded at a temperature of 37°C in NMR buffer (20 mM MES, pH 6.5, and 150 mM NaCl). A 1D ^1^H cross section shows the intensity of alanine-176. (**C**) Combined amide chemical shift differences between [*U*-^2^H,^15^N]-Skp(WT) and [*U*-^2^H,^15^N]-Skp(A108L) with bound unlabeled tOmpA. The magnitude of 2 SDs [0.053 parts per million (ppm)] is indicated by a dashed line. (**D**) Structural model of Skp(108L) with bound tOmpA. Amide groups with chemical shift changes larger than 2 SDs upon binding of tOmpA to Skp(A108L) are marked in light blue. The position of A108 is indicated by a blue circle. (**E** and **F**) 2D [^15^N,^1^H]-TROSY fingerprint spectra of [*U*-^2^H,^15^N] Skp(A108L) in the absence (E) and presence (F) of unlabeled tOmpA. Spectra were recorded at 37°C in NMR buffer. The spectral area 7.5 to 8.5 ppm in ^1^H, corresponding to disordered protein states, is indicated by gray lines. 1D ^1^H cross sections of lysine-141 in the monomeric (M) and trimeric (T) state of Skp are shown, and the relative fractions are indicated.

Having established that Skp activation comprises an equilibrium between a folded trimer and a disordered monomer, it appears relevant to understand how this equilibrium contributes to Skp chaperone activity. As a model client, we use the native client protein tOmpA, an eight-stranded transmembrane domain of OmpA. tOmpA, when bound to Skp, adopts a conformational ensemble of rapidly reorienting conformers (*15*). To investigate whether tOmpA binds to the trimeric or the monomeric state of Skp, or to both, we used an activity assay with all mutants. In a first step, we measured the chaperone activity by quantifying the amount of Skp-bound tOmpA. Intriguingly, the activity correlated with the concentration of the trimer for all Skp variants, such that, e.g., Skp(A108L) has around 50% of the Skp(WT) activity and that no chaperone activity could be detected for Skp(V117P) and Skp(A108R) (Fig. 3, A and B, and fig. S4).

**Fig. 4:**
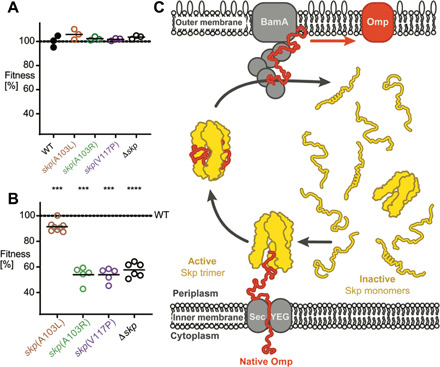
Essentiality and the functional cycle of the periplasmic chaperone Skp. (**A**) Fitness of *Salmonella* strains with various chromosomal *skp* mutations in rich lysogeny broth. Data for individual cultures and means are shown. (**B**) Fitness of *Salmonella* strains in a mouse infection model. Each circle represents data for one mouse from a total of two independent infection experiments (****P < 0.0001 and ***P < 0.001; statistical significance of difference to values for WT based on *t* test with Holm-Šídák correction for multiple comparisons). Corresponding competitive index data are shown in fig. S4. (**C**) Functional cycle of Skp. In the absence of client proteins, Skp populates the periplasm in monomeric form up to low micromolar concentrations. These partially disordered monomers are functionally inactive. An emerging Omp client at the inner membrane recruits an active trimeric chaperone from the ensemble equilibrium. Upon release of the client, trimeric Skp dissociates and the monomers enter the pool of inactive disordered conformations. See text for details.

We then selected Skp(A108L) to characterize structure and arrangement of the tOmpA-Skp(A108L) complex. First, addition of tOmpA to Skp(A108L) increases the apparent molecular mass in SEC-MALS experiments (fig. S4). Second, the 2D [^15^N,^1^H]-TROSY NMR spectra of isotope-labeled tOmpA bound to unlabeled Skp(A103L), Skp(A103R), Skp(A108L), or unlabeled Skp(WT) are highly similar (Fig. 3B and fig. S4). Because the chemical shift is a population-weighted average over the individual conformers in the tOmpA ensemble, this observation indicates that the client conformational ensemble inside the chaperone is essentially unperturbed by the local structural adaptations, resulting from the mutation A103L, A103R, or A108L. Third, a direct spectral comparison showed that the chemical shift perturbations that occur on the Skp trimeric state upon tOmpA binding are highly similar for Skp(WT), Skp(A103L), Skp(A103R), and Skp(A108L) (Fig. 3, C and D, and fig. S4). Identically to the apo state, only one set of NMR signals is present for the trimeric state, showing that the complex with tOmpA does not involve other stable oligomeric states (Fig. 4, D to G). Furthermore, for all mutants with a considerable population of the trimeric state, binding of tOmpA induces similar chemical shift perturbations, confirming a similar mode of binding (Fig. 3, C and D, and fig. S3). As a consequence, the structural description that was previously established for the Skp-tOmpA complex (*15*) can be assumed in good first-order approximation also for Skp(A108L), although the thermodynamics and kinetic of the ensemble are somewhat different (Fig. 3, B to D).

Then, we investigated the effect of tOmpA binding on the Skp monomer-trimer equilibrium at a temperature of 37°C, where Skp(A108L) is more than 80% in its monomeric state and Skp(A108R) and Skp(V117P) are completely monomeric (Fig. 3, E and F, and fig. S4). For Skp(A108L), binding of tOmpA resulted in a strong shift of the population levels from the monomeric toward the trimeric state, while no change was observed for Skp(A108R) and Skp(V117P) (Fig. 3, E and F, and fig. S4). Furthermore, for all Skp variants with considerable population of the monomeric state, the NMR signal positions of the monomeric state were not perturbed by the addition of tOmpA, confirming that there is no detectable interaction between monomeric Skp and the Omp client (fig. S3). This is an additional proof that only the structured trimer, but not the disordered monomer, has chaperone activity.

Because a bound tOmpA client is in direct contact with all three arms of Skp simultaneously (*15*), client binding contributes by avidity to the thermodynamic stability of the trimeric state of the chaperone. We quantified the difference in free energy of apo-Skp(WT) in comparison to tOmpA-Skp(WT) by a denaturation titration (fig. S4). Binding of tOmpA to Skp(WT) increased its stability by 1.7 kJ mol^−1^, corroborating the stabilization effect of the trimeric state by the binding of its client protein. Overall, the data show that monomeric, disordered Skp does not interact with the Omp client and that client binding increases the stability of the Skp trimer by avidity, thus shifting the conformational equilibrium toward the trimeric state.

### Relevance of the Skp activation mechanism for bacterial virulence

Skp is dispensable for growth of various bacterial species under rich laboratory conditions. However, bacterial pathogens such as *Yersinia* and *Salmonella* require Skp for growth in hostile host tissue. To determine whether the Skp activation mechanism that we identified is important under these physiologically relevant conditions, we engineered analogous point mutants in *Salmonella enterica* serovar Typhimurium. *Salmonella* Skp is highly homologous to *E. coli* Skp, with 91% identity (fig. S4). We selected three of the mutations for these experiments, the two mildest ones A103R and A103L, as well as V117P, and also engineered a strain with complete genetic deletion of the *skp* gene (Δ*skp*). As expected, neither the point mutants nor a full *skp* deletion affects *Salmonella* fitness in rich lysogeny broth (Fig. 4A and Table 1). We then tested the same mutants in competitive infections in a mouse typhoid fever model. In competitive infections, mice are infected with a mixture of WT and mutant strains. Plating of bacteria retrieved from spleen of these mice yields the fitness of mutants relative to the WT bacteria in each mouse. This approach reduces interindividual variance and offers higher statistical power with limited numbers of experimental animals compared to single-strain infections. The data reveal a slight but significant fitness defect of *Salmonella skp*(A103L) compared to WT and strong fitness defects for mutants *skp*(A103R) and *skp*(V117P), which are comparable to the full *skp* deletion (Fig. 4B and Table 1; competitive index data in fig. S4). These results show that already subtle perturbations of the Skp monomer-trimer equilibrium diminish Skp function in vivo and that perturbation of this equilibrium by less than an order of magnitude in *C*_0.5_ completely abolishes Skp function, rendering bacteria nonvirulent.

## DISCUSSION

In this work, we have elucidated the activation mechanism of the molecular chaperone Skp at atomic resolution. The monomer state of Skp is intrinsically disordered, with a limited residual propensity of α-helicity in the coiled-coil tentacle arms. This low inherent stability of helices α2 and α3 is particularly interesting, because they are not involved in inter-subunit contacts in the trimer structure. The formation of the head domain trimer merely fixes the positions of the end points of the α-helices in space, thus stabilizing them by reducing the conformational entropy of the unfolded state. This unique mechanism resembles a stapling of the coiled-coil helices to the barrel in the head domain. A directly related effect is being exploited in peptide chemistry to stabilize helical conformation of small peptides by a suitably chosen covalent circularization, the so-called stapled peptides (*42*). Furthermore, because the tOmpA client is in simultaneous direct contact with all three Skp subunits, its binding stabilizes and shifts the oligomer equilibrium of Skp toward the trimeric state. Last, the disordered Skp monomer does not exhibit chaperone activity.

These mechanistic insights integrate into an improved picture of the functional cycle of Skp in the bacterial periplasm (Fig. 4C). Monomeric, disordered Skp molecules populate the periplasmic space. As soon as a client protein emerges from the Sec translocase, the inactive monomers fold and assemble into a trimeric state around the unfolded client protein. Skp directly or indirectly transports the chain to the Bam complex for folding and insertion in the membrane and possibly also to DegP for degradation (*27*, *43*). The exact mechanism of client release is not understood, but besides direct migration to a higher-affinity target, one exciting possibility to enhance the release kinetics could be a destabilization of the oligomeric state of Skp or a stabilization of the monomeric state of Skp in the vicinity of the downstream receptor of the substrate. This may include negative charges on membranes or BamA (*36*, *44*, *45*). After client release, the disordered Skp monomers enter the periplasmic reservoir of individually inactive chaperones. The absence of a chaperoning activity of the monomer ensures that only Skp molecules with complete cavity bind clients, providing maximal chaperoning effect in an all-or-none fashion. At the same time, it introduces a directionality of the chaperoning effect toward the center of the cavity, avoiding spurious binding effects that would not be directed into the Skp cavity. These could potentially destabilize periplasmic proteins that are not intended client proteins. Last, the disordered nature of monomeric Skp might facilitate its import into the periplasm through the Sec complex upon its own biogenesis. Additional impact for this type of activation mechanism comes from a direct comparison to the activation mechanism of the chaperone SurA (*18*). SurA is constitutively active with just a dynamic modulation of its activity upon rotation of a domain connected by linkers to its chaperone core, i.e., its activity is only weakly regulated (*18*). Skp activity, in turn, is strongly regulated, with a switch between a completely inactive and an active state, as shown in this work. This stark difference matches a fundamental difference in function of these two periplasmic chaperones. Skp has high affinity for its client proteins and a strong tendency to prevent their folding and therefore presumably requires to be tightly regulated to avoid unspecific chaperone activity under no-stress conditions, whereas SurA binds unfolded OMPs with lower affinity while promoting their folding and therefore presumably does not require a strong regulation of its chaperone activity (*15*, *46*–*49*).

The Skp activation mechanism provides an elegant example how a chaperone can regulate its functional cycle in an environment depleted of any source of energy. For ATP-independent chaperones, only three types of activation mechanisms have so far been described: an order-to-disorder transition [Hsp33 (*10*), HdeA (*11*), and HdeB (*12*)], oligomer disassembly [sHSP (*6*) and TF (*7*–*9*)], and no or minor conformational change [Spy (*13*, *14*), HSP40 (*16*), SecB (*17*), and SurA (*18*)]. Skp is the first chaperone found to feature these activation mechanisms in the opposite direction and even combine them, i.e., by a disorder-to-order transition that is coupled to oligomerization. The high (nM)–affinity Skp has for its client proteins and the strong tendency to prevent their folding could represent a potential hazard to the cell (*15*, *49*). The coupled folding and oligomerization mechanism ensures that holdase function is only present in the trimer where it is geometrically oriented only toward the chaperone cavity. Under nonstressed conditions, Skp exists as an inactive disordered monomer with a minor population of active folded trimer to avoid detrimental effect for the cells. At the opposite, under stress conditions, up-regulation of the Skp concentration and binding to client proteins shift the equilibrium toward the trimeric folded active state, protecting the cells by preventing aggregation of unfolded protein. While most chaperones use strategies to cover a preexisting client binding site in their inactive state, Skp has thus evolved a more extreme mechanism where the client binding area exists only in the active state. This strong regulation allows the tight control of Skp activity while providing at the same time a fast mechanism for client release upon dissociation into the disordered monomeric state. The chaperone activity of Skp is thus regulated in dynamic response to chaperone concentration and client availability.

## MATERIALS AND METHODS

### Cloning, expression, and purification of Skp

Skp, lacking its signal sequence, was cloned from genomic DNA through Nde I and Xho I into the pET28b expression vector (Novagen) containing a thrombin-cleavable N-terminal His_6_-tag (*15*). Skp was expressed in BL21-(λ DE3)-Lemo cells [New England Biolabs (NEB)] transformed with the Skp plasmid and grown at 37°C in M9 minimal medium containing kanamycin (30 mg/ml) to OD_600_ (optical density at 600 nm) = 0.6, and then the expression was induced by adding 0.4 mM isopropyl-β-d-thiogalactopyranoside (IPTG) at 25° for 12 hours. Uniformly [^2^H, ^13^C, ^15^N]-labeled protein was prepared by growing cells in D_2_O-based M9 minimal medium, with 1 g of ^15^NH_4_Cl and 2 g of [*U*-^13^C,^2^H] glucose per liter of medium. Cells were harvested by centrifugation at 5000*g* for 20 min. The pellet was resuspended in 20 ml of lysis buffer A per liter of culture [20 mM tris (pH 7.5), 500 mM NaCl, deoxyribonuclease (DNase) (0.01 mg/ml), ribonuclease (RNase) (0.02 mg/ml), and inhibitor cocktail (cOmplete EDTA-free protease inhibitor; Roche)]. Cell lysis was performed using a microfluidizer (Microfluidics) for three cycles at 4°C. The soluble bacterial lysate was separated from cell debris and other components by centrifugation at 14,000*g* for 60 min and loaded onto a Ni-NTA (nitrilotriacetic acid) column (Qiagen). Skp eluted at 250 mM imidazole concentration and was dialyzed against buffer [20 mM tris (pH 7.5) and 500 mM NaCl] overnight to remove the imidazole. In a final step, a size exclusion chromatography (Superdex-200 16/600 PG) step was applied to further purify the proteins and adjust the protein to its final buffer [20 mM MES (pH 6.5) and 150 mM NaCl]. Note that the His_6_-tag was consistently not cleaved from all Skp constructs, because in both our hands and published work by others (*37*), the presence of the His_6_-tag was found to not change the monomer-trimer equilibrium constant and because monomeric, disordered Skp was found to be sensitive to proteolytic degradation. Afterward, Skp was concentrated by ultrafiltration and stored at −20°C until use. Final yield of purified protein was 25 mg for Skp(WT) and mutants per liter of deuterated M9 minimal medium.

### Cloning, expression, and purification of tOmpA

The transmembrane domain of OmpA (residues 1 to 177) was cloned through Nco I and Xho I into the pET28b expression vector without any affinity tag and lacking its signal sequence (*15*). BL21-(λ DE3)-Lemo cells (NEB) were transformed with the tOmpA expression plasmid and grown at 37°C in medium containing kanamycin (30 μg/ml) to OD_600_ = 0.8. Expression was induced by 1 mM IPTG. Cells were harvested 4 hours after induction and resuspended in 20 ml of buffer B per liter of culture (20 mM tris-HCl and 5 mM EDTA, pH 8.5). Cell lysis was performed using a microfluidizer (Microfluidics) for three cycles at 4°C. Purification from inclusion bodies was done as described (*50*). The ion-exchange elution fractions containing tOmpA were pooled and dialyzed against buffer B. The precipitate was resuspended in 6 M Gdm/HCl and stored at −20°C until usage. Final yield of purified protein was 50 mg of tOmpA per liter of deuterated M9 minimal medium.

### Mutagenesis, expression, and purification of Skp mutants

The QuikChange II mutagenesis protocol (Stratagene) was used to introduce the mutations A108L, A108R, A103L, A103R, or V117P into Skp. Polymerase chain reaction (PCR) primers (Table 2) were obtained from Microsynth. The expression and purification of the mutant proteins was performed as described for the WT proteins. The final yield of purified mutants was similar to WT.

**Table 2:** List of oligonucleotide primers used in this study.

**Oligonucleotides**	**Sequences**
*Skp*(A103L) forward	5′-CTGGTTACTCGTATCCAGACTCTAGTGAAATCCGTTGCCAACAGC-3′
*Skp*(A103L) backward	5′-GCTGTTGGCAACGGATTTCACTAGAGTCTGGATACGAGTAACCAG-3′
*Skp*(A103R) forward	5′-CAAACTGGTTACTCGTATCCAGACTCGTGTGAAATCCGTT-3′
*Skp*(A103R) backward	5′-AACGGATTTCACACGAGTCTGGATACGAGTAACCAGTTTG-3′
*Skp*(A108L) forward	5′-AGATCGATATCCTGGCTGTTTAGAACGGATTTCACAGCAGTCTGG-3′
*Skp*(A108L) backward	5′-CCAGACTGCTGTGAAATCCGTTCTAAACAGCCAGGATATCGATCT-3′
*Skp*(A108R) forward	5′-CGATATCCTGGCTGTTGCGAACGGATTTCACAGCAG-3′
*Skp*(A108R) backward	5′-CTGCTGTGAAATCCGTTCGCAACAGCCAGGATATCG-3′
*Skp*(V117P) forward	5′-ACAGCCAGGATATCGATCTGGTTCCTGATGCAAACGCC-3′
*Skp*(V117P) backward	5′-GGCGTTTGCATCAGGAACCAGATCGATATCCTGGCTGT-3′

### Mouse infection model

*Salmonella* strains used in this study were based on *S. enterica* serovar Typhimurium SL1344 *hisG xyl* (*51*, *52*). *Salmonella* mutants with gene deletions were obtained by two consecutive single crossovers with positive selection for resistance to kanamycin and negative selection for levansucrase-mediated sensitivity to sucrose. *Salmonella* was grown in lysogeny broth containing NaCl (5 g/liter; “Lennox LB”). Each strain was transformed with a low-copy plasmid expressing a distinct fluorescent protein (mtagBFP2, mNeonGreen, YPet, or mCherry). These plasmids have no impact on in vivo fitness (*53*, *54*). All animal experiments were approved (license 2239, Kantonales Veterinäramt Basel) and performed according to local guidelines (Tierschutz-Verordnung, Basel) and the Swiss animal protection law (Tierschutz-Gesetz). Eight 10- to 16-week-old female BALB/c mice (Charles River Laboratories) were infected by tail vein injection of mixtures containing WT *Salmonella* and different combinations of three mutants with about 1000 colony-forming units (CFU) each per strain. The exact inoculum size for each strain was determined by plating. After 4 days, mice were euthanized with carbon dioxide and Triton X-100 detergent–treated spleen homogenates were prepared as described previously (*55*). Total *Salmonella* loads were determined by plating dilution series on agar plates. Mutant-to-WT ratios were determined by flow cytometry counting of bacterial cells falling into gates indicative for the various fluorescent proteins using optical filters (*55*). Fitness was calculated as log_2_(FI), with FI corresponding to the fold increase starting from the initial spleen colonization [around 20% of the inoculum (*56*)] to the final spleen load for each strain. The relative fitness value of co-administered WT *Salmonella* was set to 100%. We also determined the more commonly used readout “competitive index” by dividing the output ratio (mutant/WT) by the inoculum ratio (mutant/WT).

### Complex assembly and aggregation assay

Complex assembly was carried out following a modified version of the protocol published by Burmann *et al.* (*15*). A 1.5 M excess of denatured tOmpA was added to Skp(WT) or mutants in 20 ml of assembly buffer [20 mM MES (pH 6.5) and 150 mM NaCl] in a dropwise fashion under continuous stirring. The solution was then stirred for another 1 hour to ensure saturation of the chaperones. After centrifugation at 10,000*g* for 30 min, the supernatant fraction, containing the Skp-tOmpA complexes, was separated from the pellet, containing the precipitated tOmpA. The supernatant was exchanged by ultrafiltration to NMR buffer [20 mM MES (pH 6.5) and 150 mM NaCl], and after concentration, the volume was adjusted to 250 μl. The chaperone activity of Skp(WT) and mutant was determined by quantifying the NMR signals in 2D [^15^N,^1^H]-TROSY spectra of [*U*-^2^H,^15^N]-tOmpA bound to unlabeled Skp. Control sample of [*U*-^2^H,^15^N]-tOmpA in NMR buffer was prepared following the reference protocol, showing that, in the absence of the functional Skp(WT), less than 2% of [*U*-^2^H,^15^N]-tOmpA signals were observed in comparison to [*U*-^2^H,^15^N]-tOmpA bound to Skp(WT).

### NMR spectroscopy

All NMR experiments for Skp-Omp complexes were performed in NMR buffer [20 mM MES (pH 6.5) and 150 mM NaCl]. The experiments were recorded at the specified temperature on a Bruker AscendII 700 MHz or Avance 800 MHz spectrometer running Topspin 3.0 and equipped with a cryogenically cooled triple-resonance probe. For the sequence-specific backbone resonance assignment of [*U*-99% ^2^H, ^13^C, ^15^N]-*Skp*(A108L), the following NMR experiments were recorded at 37°C: 2D [^15^N,^1^H]-TROSY, 3D TROSY-HNCA, 3D TROSY-HNCACB, 3D TROSY-HNCO, and 3D TROSY-HN(CA)CO. NMR data were processed with nmrPipe (*57*) and analyzed with CARA and ccpnmr (*58*). Secondary chemical shifts were calculated relative to the random-coil values of Kjaergaard and Poulsen (*59*). For the backbone assignment of the unfolded [*U*-^2^H,^15^N,^13^C]-Skp(WT), automated projection spectroscopy (APSY) experiments were recorded in NMR buffer [20 mM MES (pH 6.5) and 150 mM NaCl] containing 8 M urea at 15°C. The 5D APSY-HNCOCACB (*60*) was recorded with 54 transients for Skp, two scans per transient, 0.7-s recycle delay, and 1024 × 150 complex points in the direct and indirect dimensions. The 4D APSY-HNCACB (*60*) was recorded with 46 transients, two scans per transient, 0.7-s recycle delay, and 1024 × 180 complex points in the direct and indirect dimensions, respectively. The GAPRO (geometric analysis of projections) (*60*) analysis of the projection spectra was carried out with Δυ = 5.0 Hz, *R*_min_ = 15.0 Hz, *S*/*N* = 7.0, and *S*_min,1_ = *S*_min,2_ = 8 for the 5D APSY-HNCOCACB and with Δυ = 5.0 Hz, *R*_min_ = 15.0 Hz, *S*/*N* = 10.0, and *S*_min,1_ = *S*_min,2_ = 15 for the 4D APSY-HNCACB. As the signals for glycine residues within the 4D APSY-HNCACB and the signals of residues succeeding glycines within the 5D APSY-HNCOCAB have a different sign than the other resonances, the GAPRO algorithm was run twice for positive and negative peaks, respectively, and the two resulting peak lists were combined. The combined peak lists were assigned by using the newest version of the MATCH algorithm within the UNIO´10 software package, yielding a 65% complete assignment for Skp. By using a conventional 3D TROSY-HNCACB experiment, complete backbone assignment for Skp could be obtained. NMR data were processed using PROSA (*61*) and analyzed with CARA and XEASY. Combined chemical shift differences of the amide resonances in 2D [^15^N,^1^H]-TROSY spectra were calculated as

ΔδHN=(Δδ(H1))2+(0.2·Δδ(N15))2 (1)

### Size-exclusion chromatography coupled to multi-angle light scattering

SEC-MALS measurements of Skp were performed at 25°C in NMR buffer [20 mM MES (pH 6.5) and 150 mM NaCl] using a GE Healthcare Superdex-200 Increase 10/300 GL column on an Agilent 1260 high-performance liquid chromatography. Elution was monitored using an Agilent multi-wavelength absorbance detector (data collected at 280 and 254 nm), a Wyatt Heleos II 8+ multiangle light-scattering detector, and a Wyatt Optilab rEX differential refractive index detector. The column was equilibrated overnight in the running buffer to obtain stable baseline signals from the detectors before data collection. Inter-detector delay volumes, band broadening corrections, and light-scattering detector normalization were calibrated using an injection of bovine serum albumin solution (2 mg/ml; ThermoPierce) and standard protocols in ASTRA 6. Weight-averaged molar mass, elution concentration, and mass distributions of the samples were calculated using the ASTRA 6 software (Wyatt Technology).

### Differential scanning calorimetry

DSC data were acquired using a Microcal VP-Capillary DSC instrument (Malvern Panalytical, Malvern UK) at a Skp trimer concentration of 24.4 μM (i.e., 73 μM concentration in terms of monomer). After centrifugation, protein concentration was determined by ultraviolet spectrophotometry using a molar extinction coefficient of 4470 M^−1^ cm^−1^ at 280 nm for the trimer and correcting for minor scattering contributions to apparent absorbance. The Skp sample was scanned from 15° to 105°C at a scan rate of 1°C/min, and data points were acquired at 0.1°C increments. Multiple buffer versus buffer scans, performed before the sample scan to establish the instrumental heat capacity baseline, were averaged and subtracted from the sample scan data, which were then normalized to excess molar heat capacity using the trimer concentration. Attempts to fit the complex thermogram with standard models of oligomer dissociation and denaturation proved unsuccessful, so Δ*C*_p_ for folding was estimated from the difference between the slopes of the excess molar heat capacity in low- and high-temperature regions (the apparent pre- and post-transition baselines), fitted by linear regression, and extrapolated to the temperature of interest.

### Description of the monomer-trimer equilibrium

The chemical equilibrium between trimeric and monomeric Skp can be described by the reaction

$$3\;S_{m}⇋S_{t}$$ (2)

where the equilibrium constant *L*_13_ is given by

$$L_{13}=\frac{\left[S_{t}\right]}{{\left[S_{m}\right]}^{3}}$$ (3)

where [*S_t_*] and [*S_m_*] are the molar concentrations of Skp trimers and free Skp monomers, respectively, and *L*_13_ has units of M^−2^. For this equilibrium, the concentration of trimer [*S_t_*] as a function of total Skp [*S*_0_] is given by Sandlin *et al.* (*37*)

$$[S_{t}]([S_{0}])=\frac{\left[S_{0}\right]}{3}+\frac{\delta }{{\left(\sqrt{\beta^{2}-\xi^{3}}+\beta \right)}^{\frac{1}{3}}}+{\left(\sqrt{\beta^{2}-\xi^{3}}+\beta \right)}^{\frac{1}{3}}$$ (4)

where δ, ξ, and β are given by

$$\delta =\frac{9L_{13}{\left[S_{0}\right]}^{2}+1}{L_{13}}$$ (5)

$$\xi =\frac{{\left[S_{0}\right]}^{2}}{9}-\frac{\delta }{81}$$ (6)

$$\beta =\frac{{\left[S_{0}\right]}^{3}}{18}-\frac{[S_{0}]\delta }{162}$$ (7)

The fraction of total Skp protein that is trimeric at any total Skp concentration equals

$$f_{S_{t}}=\frac{3[S_{t}]}{\left[S_{0}\right]}$$ (8)

and the fraction of total Skp protein that is monomeric equals

$$f_{S_{m}}=1-f_{S_{t}}$$ (9)

### SEC-MALS quantification of the trimeric and monomeric population

In SEC-MALS experiments in equilibrium situations, the detected molar mass represents the concentration-weighted average mass of the species involved

$$M_{w}=\frac{\Sigma (c_{i}M_{i})}{\Sigma (c_{i})}$$ (10)

where *c_i_* is the mass concentration and *M_i_* is the molar mass of the *i*th species. Therefore, for the monomer-trimer equilibrium, by comparison with the limits for the completely monomeric or trimeric species, the weight-averaged mass reports directly on the fractional populations as

$$f_{S_{m}}=\frac{M_{\text{obs}}-M_{S_{t}}}{M_{S_{m}}-M_{S_{t}}}$$ (11)

where *M*_obs_ is the detected weight-averaged mass, and *M_S_m__* and *M_S_t__* are the detected masses of the completely monomeric and trimeric state, respectively.

### NMR quantification of the trimeric and monomeric population

For the estimation of the population of monomeric and trimeric states for the WT and mutants, the residue lysine-141 was chosen, because its signals are well resolved in each state and it is located in a nonstructured, locally flexible region in the trimer. The fractions were estimated by calculating the ratio of the intensity of the signals in the monomeric and trimeric state according to the equation

$$f_{S_{m}}=\frac{I_{S_{m}}}{I_{S_{m}}+I_{S_{t}}}$$ (12)

where *I_S_m__* and *I_S_t__* are the intensity of the residue lysine-141 in the monomeric and trimeric state, respectively. Similarly, for the denaturation titration, the fractions of folded and unfolded Skp were determined from the signals of residue lysine-141, and for each titration point, Δ*G* was calculated assuming a two-state model according to the equation

$$\Delta G=-R\cdot T\cdot \text{ln}\frac{f_{S_{t}}}{f_{S_{m}}}$$ (13)

The data were fitted by linear regression, and Δ*G* was extrapolated to a concentration of 0 M urea.

## Supplementary Material

abc5822_SM.pdf

## SUPPLEMENTARY MATERIALS

Supplementary material for this article is available at http://advances.sciencemag.org/cgi/content/full/6/43/eabc5822/DC1

## Appendix

View/request a protocol for this paper from *Bio-protocol*.
