# Supplementary material for: Regulation of chaperone function by coupled folding and oligomerization
Source: Sci Adv. 2020 Oct 21;6(43):eabc5822. doi: 10.1126/sciadv.abc5822 (PMC7577714; doi:10.1126/sciadv.abc5822)
Supplement: abc5822_SM.pdf [file abc5822_SM.pdf]

Science Advances
AAAS

advances.sciencemag.org/cgi/content/full/6/43/eabc5822/DC1

Supplementary Materials for

Regulation of chaperone function by coupled folding and oligomerization

Guillaume Mas, Björn M. Burmann, Timothy Sharpe, Beatrice Claudi, Dirk Bumann, Sebastian Hiller*

*Corresponding author. Email: sebastian.hiller@unibas.ch

Published 21 October 2020, *Sci. Adv.* **6**, eabc5822 (2020)
DOI: 10.1126/sciadv.abc5822

This PDF file includes:

Figs. S1 to S4

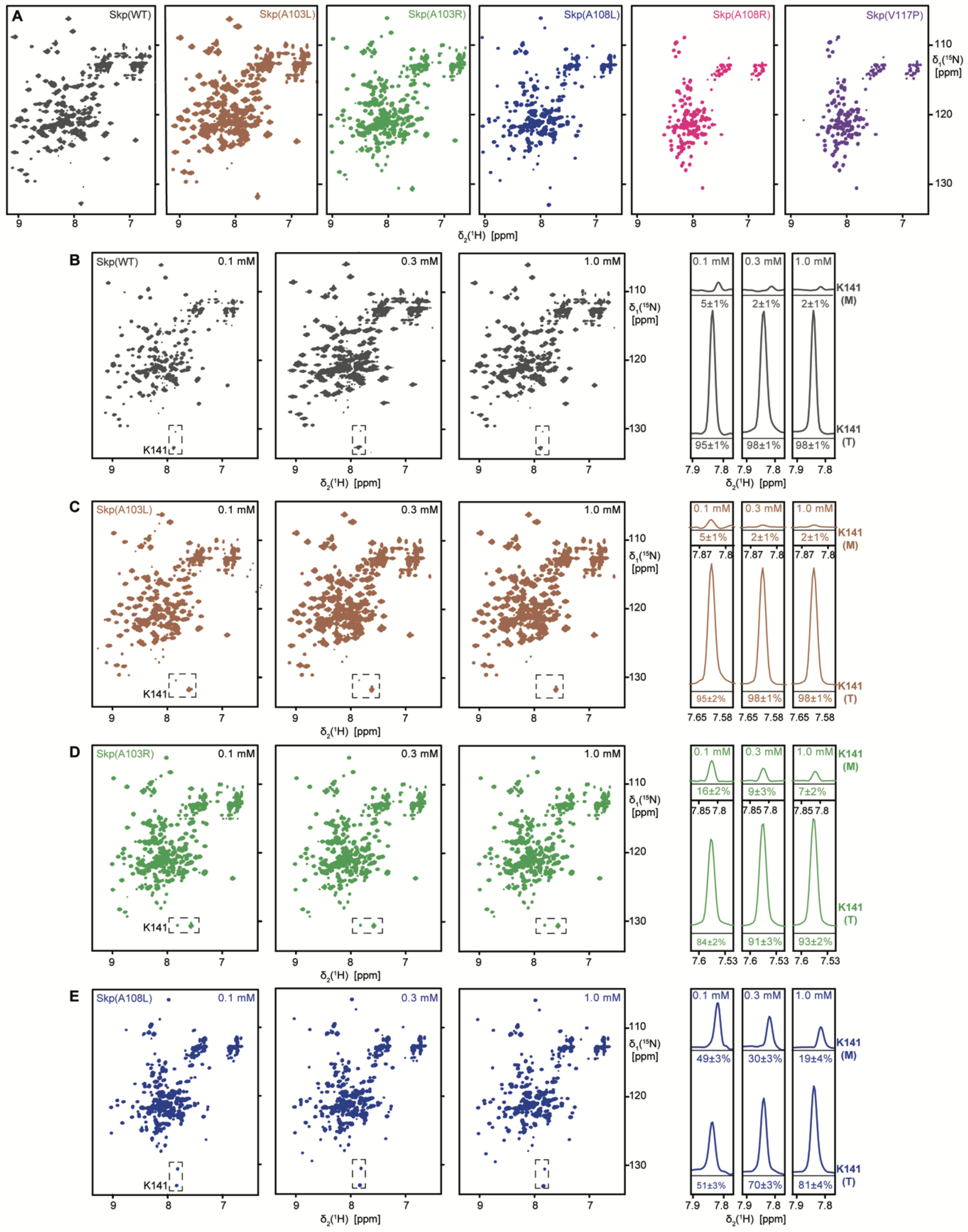

Fig. S1. Quantification of the Skp monomer–trimer equilibrium by NMR spectroscopy. (**A**) 2D [^{15}N,^{1}H]-TROSY spectra of [U-^{2}H,^{15}N] Skp(WT) (dark grey), Skp(A103L) (brown), Skp(A103R) (green), Skp(A108L) (blue), Skp(A108R) (magenta) and Skp(V117P) (purple) at a concentration of 1 mM, recorded at 25 °C in NMR buffer (20 mM MES, pH 6.5, 150 mM NaCl) at 700 MHz. (**B–E**) 2D [^{15}N,^{1}H]-TROSY spectra of [U-^{2}H,^{15}N] Skp(WT) (**B**),

Skp(A103L) (**C**), Skp(A103R) (**D**) and Skp(A108L) (**E**) at concentrations of 0.1, 0.3 and 1 mM, recorded at 25 °C in NMR buffer at 700 MHz. For each construct, the position of lysine 141 is highlighted by a dashed box. On the right-hand side, 1D ^{1}H cross sections of the 2D spectra are shown, taken at the position of lysine 141 in either the monomeric (M) or trimeric (T) Skp. The relative fractions of monomer and trimer are given below the peaks.

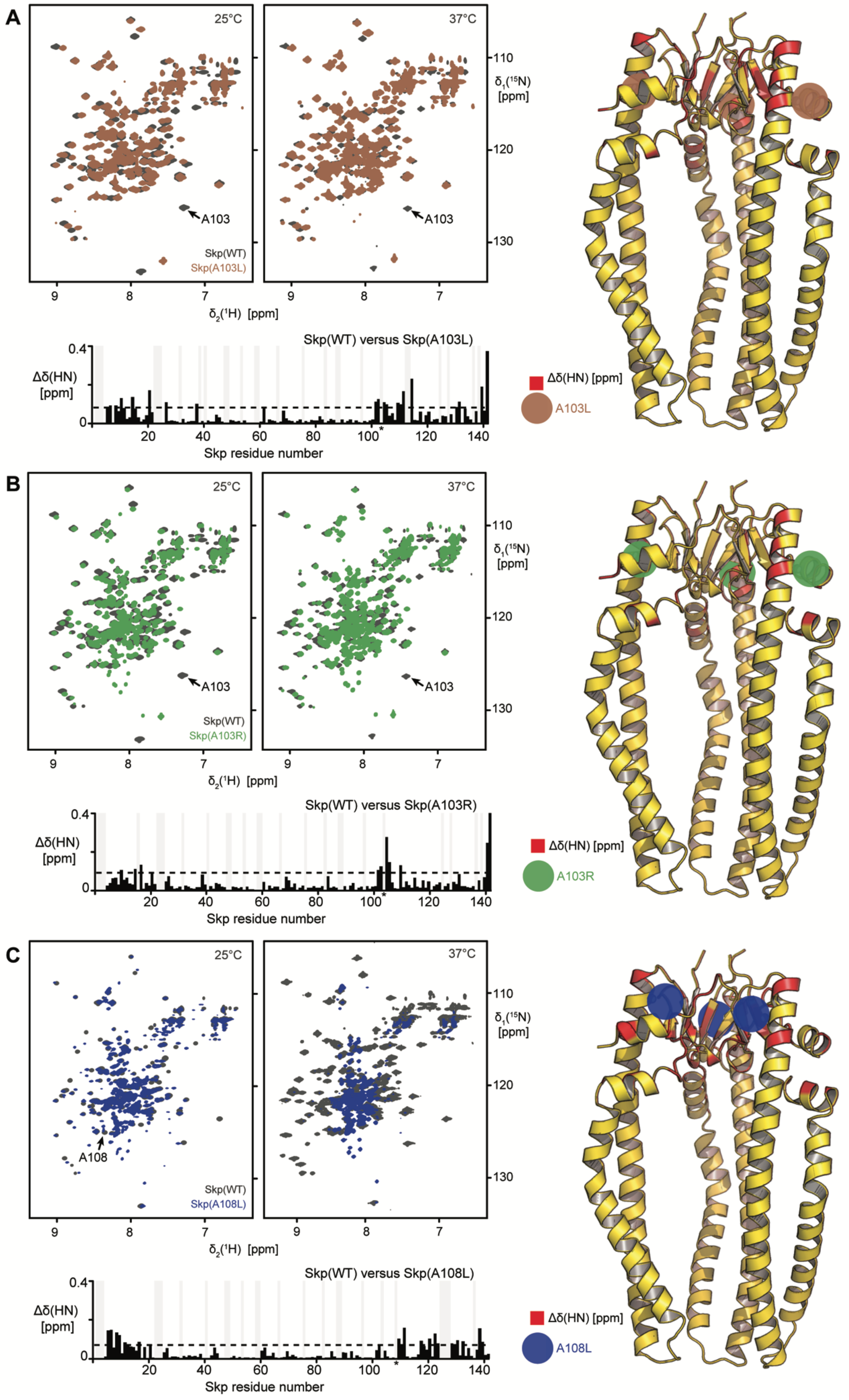

A
25°C
37°C
A103
Skp(WT)
Skp(A103L)
δ1(15N) [ppm]
δ2(1H) [ppm]
Skp(WT) versus Skp(A103L)
Δδ(HN) [ppm]
Skp residue number
A103L
B
A103
Skp(WT)
Skp(A103R)
Skp(WT) versus Skp(A103R)
A103R
C
A108
Skp(WT)
Skp(A108L)
Skp(WT) versus Skp(A108L)
A108L

Fig. S2. Comparison of Skp variants by NMR spectroscopy. (**A**) Overlay of 2D [^{15}N,^{1}H]-TROSY spectra of [*U*-^{2}H,^{15}N] Skp(WT) (dark grey) with Skp(A103L) (brown) at a concentration of 1 mM, recorded at 25 °C or 37 °C in NMR buffer (20 mM MES, pH 6.5, 150 mM NaCl) at 700 MHz. Below the 2D spectra, the combined amide chemical shift differences between Skp(WT) and Skp(A108L) at 25 °C are plotted against the Skp amino acid residue number. The magnitude of two standard deviations is indicated by a horizontal dashed line. Grey bars denote unassigned residues. On the right-hand side, all residues with amide chemical shift differences larger than two s.d. are marked in red on the Skp structure, and the position of the mutation site is shown by a colored circle. (**B**) As in **A**, but for Skp(A103R) (green) (**C**) As in **A** and **B**, but for Skp(A108L) (blue).

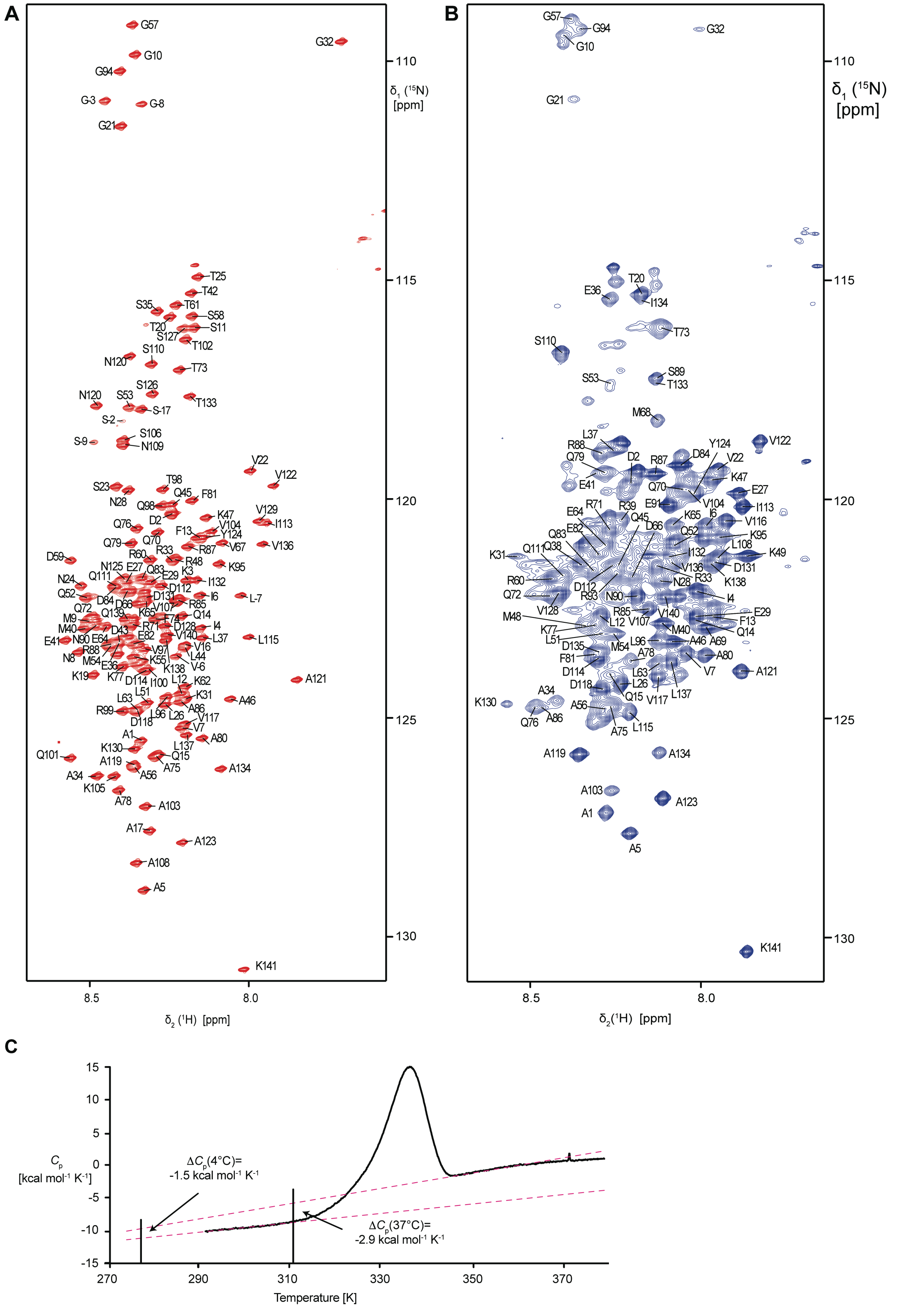

Fig. S3. NMR assignment of the Skp monomer and thermodynamic stability of the Skp trimeric. (**A**) 2D $[^{15}N,^{1}H]$-HSQC spectrum of 1 mM $[U\text{-}^{2}H,^{13}C,^{15}N]$–Skp(WT) (red) in

denaturing buffer (20 mM MES, pH 6.5, 150 mM NaCl, 8 M urea]. The sequence-specific resonance assignment obtained from multidimensional APSY and a 3D HNCACB triple resonance experiment is indicated. (**B**) 2D [^{15}N,^{1}H]-TROSY spectrum of 1 mM [*U*-^{2}H,^{15}N,^{13}C] Skp(A108L), recorded at a temperature of 37 °C in NMR buffer at 700 MHz. The sequence-specific resonance assignments of the monomeric state, obtained from 3D triple resonance experiments, are indicated. (**C**) Differential scanning calorimetry thermogram of Skp(WT) in 20 mM Tris (pH 8.0), 150 mM NaCl. The difference in excess molar heat capacity at constant pressure between the folded and the unfolded state, ΔC_p, was approximated as the difference between the pre- and post-transition baselines, fitted by linear regression and extrapolated to the temperature of interest (dashed pink lines). ΔC_p at 4 °C and 37 °C are indicated by arrows.

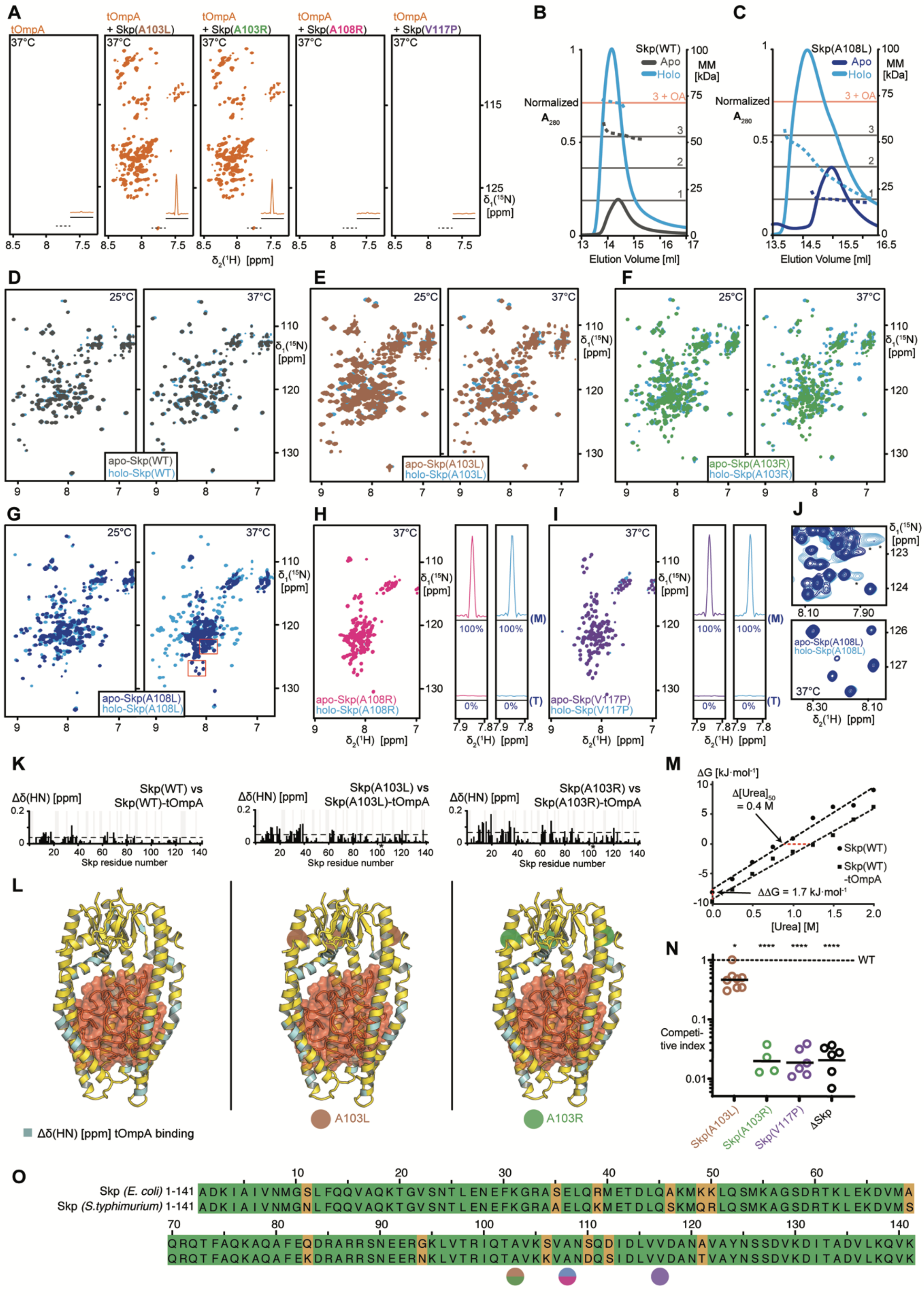

Fig. S4. Effect of client binding to Skp variants. (**A**) 2D [^{15}N,^{1}H]-TROSY fingerprint spectra of the soluble fraction of [*U*-^{2}H,^{15}N]-tOmpA added to unlabeled Skp variants or absence of Skp as control, as indicated. Spectra were recorded in NMR buffer at 37 °C after removal of

precipitated tOmpA. On the bottom right of each spectrum, a 1D ^{1}H cross section taken at the position of alanine 176 is shown. (**B**) SEC elution profiles (solid lines, left axis) and MALS apparent molecular mass (dotted lines, right axis) for apo-Skp(WT) and its complex with tOmpA (cyan). Grey horizontal lines indicate the molecular weight corresponding to one, two and three monomers of Skp. The red horizontal line indicates the molecular weight of a Skp trimer in complex with tOmpA. (**C**) As **B**, but with Skp(A108L). (**D–G**) Overlay of 2D [^{15}N,^{1}H]-TROSY spectra of [*U*-^{2}H,^{15}N] apo-Skp (dark grey) and [*U*-^{2}H,^{15}N] Skp with bound unlabelled tOmpA (cyan), recorded at 25 °C (left panel) or 37 °C (right panel) in NMR buffer at 700 MHz; (**D**) Skp(WT); (**E**) Skp(A103L); (**F**) Skp(A103R); (**G**) Skp(A108L). For Skp(A108L), the areas indicated by a red square correspond to the zoom in **J**. (**H**) Overlay of 2D [^{15}N,^{1}H]-TROSY spectra of [*U*-^{2}H,^{15}N] apo-Skp(A108R) (magenta) and [*U*-^{2}H,^{15}N] Skp(A108R) after addition of unlabelled tOmpA, and removal of the precipitated tOmpA (cyan), recorded at 37°C in NMR buffer at 700 MHz. 1D ^{1}H cross sections taken at the position of lysine 141 in monomeric (M) and trimeric (T) Skp are presented on the right of the spectra with the respective fractions indicated. (**I**) As in **H**, but for Skp(V117P). (**J**) Zooms on the 37 °C spectra in panel **G**. Signals highlighted by an asterisk correspond to the folded trimeric state. (**K**) Combined amide chemical shift differences between apo Skp and Skp in Skp–tOmpA complexes for Skp(WT), Skp(A103L), and Skp(A103R), as indicated. The magnitude of two standard deviations is indicated by a dashed line. (**L**) Structural models of Skp(WT), Skp(A103L) and Skp(A103R) with bound tOmpA. Amide groups with chemical shift changes larger than two s.d. upon binding of tOmpA to are marked in light blue. The position of the mutations A103L and A103R are indicated by a brown and green circle, respectively. (**M**) Urea denaturation of apo-Skp(WT) (black circles) and Skp(WT)–tOmpA (black squares). The dashed lines represent linear fits to the data. (**N**) Competitive indices of *Salmonella* strains vs. wild-type (output ratio mutant/wild-type divide by the input ratio mutant/wild-type) in a mouse infection model. Each circle represents data for one mouse from a total of two independent infection experiments (****, $P< 0.0001$; ***, $P< 0.001$; *, $P< 0.05$; statistical significance of difference to values for wild-type *Salmonella* based on t-test with Holm-Šídák correction for multiple comparisons). (**O**) Sequence alignment of Skp from *E. coli* and *Salmonella typhimurium*. Identical and different residues are green and orange, respectively. The positions of mutations used in this work are indicated below the alignment.
